# Polariton hyperspectral imaging of two-dimensional semiconductor crystals

**DOI:** 10.1038/s41598-019-50316-8

**Published:** 2019-09-24

**Authors:** Christian Gebhardt, Michael Förg, Hisato Yamaguchi, Ismail Bilgin, Aditya D. Mohite, Christopher Gies, Matthias Florian, Malte Hartmann, Theodor W. Hänsch, Alexander Högele, David Hunger

**Affiliations:** 1 Fakultät für Physik, Ludwig-Maximilians-Universität, Schellingstraße 4, 80799 München, Germany; 2 Max-Planck-Institut für Quantenoptik, Hans-Kopfermann-Str. 1, 85748 Garching, Germany; 3 Fakultät für Physik, Munich Quantum Center, and Center for NanoScience (CeNS), Ludwig-Maximilians-Universität, Geschwister-Scholl-Platz 1, 80539 München, Germany; 4 3MPA-11 Materials Synthesis and Integrated Devices, Materials Physics and Applications Division, Los Alamos National Laboratory (LANL), Los Alamos, New Mexico 87545 USA; 5 Institut für Theoretische Physik, Universität Bremen, 28334 Bremen, Germany; 6 Physikalisches Institut, Karlsruher Institut für Technologie, Wolfgang-Gaede-Str.1, 76131 Karlsruhe, Germany

**Keywords:** Microresonators, Polaritons, Quantum optics, Fluorescence spectroscopy, Polaritons

## Abstract

Atomically thin crystals of transition metal dichalcogenides (TMDs) host excitons with strong binding energies and sizable light-matter interactions. Coupled to optical cavities, monolayer TMDs routinely reach the regime of strong light-matter coupling, where excitons and photons admix coherently to form polaritons up to room temperature. Here, we explore the two-dimensional nature of TMD polaritons with scanning-cavity hyperspectral imaging. We record a spatial map of polariton properties of extended WS_2_ monolayers coupled to a tunable micro cavity in the strong coupling regime, and correlate it with maps of exciton extinction and fluorescence taken from the same flake with the cavity. We find a high level of homogeneity, and show that polariton splitting variations are correlated with intrinsic exciton properties such as oscillator strength and linewidth. Moreover, we observe a deviation from thermal equilibrium in the resonant polariton population, which we ascribe to non-Markovian polariton-phonon coupling. Our measurements reveal a promisingly consistent polariton landscape, and highlight the importance of phonons for future polaritonic devices.

## Introduction

Exciton polaritons can enable novel photonic elements such as ultra-low threshold lasers^1,2^, Bose-Einstein condensates^3^, or quantum nonlinear optical elements^4^. Atomically thin crystals of TMDs offer a particularly promising platform to study and harness exciton polaritons due to a strong exciton binding energy^5,6^ and a large oscillator strength^7–9^. Both properties in concert have enabled the demonstration of exciton polaritons at room temperature^10–12^ and under cryogenic conditions^13–15^. Owing to their unique spin-valley degrees of freedom inherited from the non-centrosymmetric host crystal with strong spin-orbit effects^16^, TMD polaritons could enable novel photonic devices with topological properties in accordingly structured two-dimensional photonic and excitonic landscapes^17^. First demonstrations of long-range polariton propagation in multi-layer waveguides^18,19^ are a first indication of this potential.

It remains an important task to understand the conditions that govern polariton properties, and to realize large-scale systems that are useful also for such advanced devices. Elevated temperature and the crystals’ two-dimensional geometry constitute an environment that strongly influences polariton properties. Variation of both intrinsic defect concentrations and of the dielectric surrounding can lead to a significant spatial variation of the materials optical properties. However, so far, experiments studying monolayer exciton polaritons were limited to isolated locations, leaving the materials 2D nature widely unexplored. Also, *in situ* complementary measurements on the spatial variation of absorption and fluorescence of the native excitons are still lacking. In this work we reveal spatial variations and environmental influences on polaritons by hyperspectral scanning cavity microscopy in the strong coupling regime^20^. We complement this data with cavity-enhanced photoluminescence and extinction microscopy^21^, which allows us to identify clear correlations of the polariton splitting with intrinsic excitonic properties such as oscillator strength and linewidth, and extrinsic effects such as dielectric screening. Enabled by the tunability of our microcavity, we can furthermore perform spectroscopy of the polariton-phonon coupling. We observe a polariton population with significant deviations from thermal equilibrium for specific polariton splittings which coincide with peaks in the phonon density of states, suggesting a resonant, non-Markovian polariton-phonon coupling^22–25^. Our measurement technique reveals a promisingly homogeneous two-dimensional polariton landscape with variations that we can directly trace back to intrinsic and extrinsic influences, and with strong impact from polariton-phonon coupling, providing important insight for future polaritonic devices.

## Results

### Cavity and WS_2_ spectroscopy

**Figure 1:**
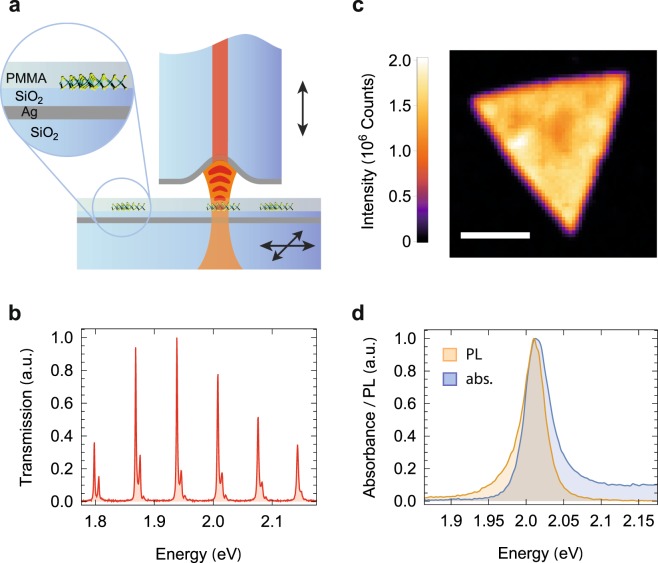
Experimental setup and characterisation. (**a**) A fiber-based microcavity couples to monolayer WS_2_ covered with a thin film of PMMA on top of a silver (Ag) mirror capped with a dielectric spacer (SiO_2_). The planar mirror is mounted on a 3D nanopositioning stage to enable raster-scanning of the sample through the cavity mode and coarse tuning of the mirror separation. The cavity length and thus its resonance frequency can be fine-tuned by a piezo actuator moving the fiber. (**b**) White light transmission spectrum of the empty cavity for a large mirror separation *d* = 10.5 *μ*m. (**c**) Confocal PL map of a WS_2_ flake on the mirror (scale bar 10 *μ*m). (**d**) Confocal PL (orange) and cavity-assisted absorption (blue) spectra recorded on a typical position of a WS_2_ flake on the mirror.

Our experimental platform is a fiber-based Fabry-Perot microcavity^26^ consisting of a laser-machined optical fiber serving as a micromirror and a planar mirror with monolayer flakes of WS_2_ synthesized by chemical vapor deposition (CVD) and covered with a thin film of PMMA (see Fig. 1a and Methods). All experiments are performed at room temperature. Away from the flakes, the transmission of the bare cavity shown in Fig. 1b features the characteristics of a stable Fabry-Perot resonator with Hermite-Gaussian eigenmodes that exhibits a strong main resonance and a blue-detuned weak resonance stemming from higher transverse modes. The empty cavity has a finesse of 40 at the exciton energy of 2.01 eV, leading to a cavity-length dependent linewidth *κ* = 51/*q* meV, where *q* is the longitudinal mode order.

In the first experimental step, we characterized the CVD-grown WS_2_ monolayer flakes on the macroscopic mirror with confocal microscopy and spectroscopy and found extended triangular flakes with bright photoluminescence (PL) as in Fig. 1c. We observe PL spectra as shown in Fig. 1d with a full-width-half-maximum (FWHM) linewidth *γ* = 33 meV, typical for monolayer WS_2_^27^ and indicative of only minor inhomogeneous broadening. For the same position, we also measured the absorption spectrum of Fig. 1d by cavity-enhanced spectroscopy at large mirror separation where the exciton-photon coupling is weak (see methods). The absorption spectrum matches the center energy and the linewidth of the emission, but shows additional finite background absorption at the higher energy side of the resonance.

### Tunable strong coupling

**Figure 2:**
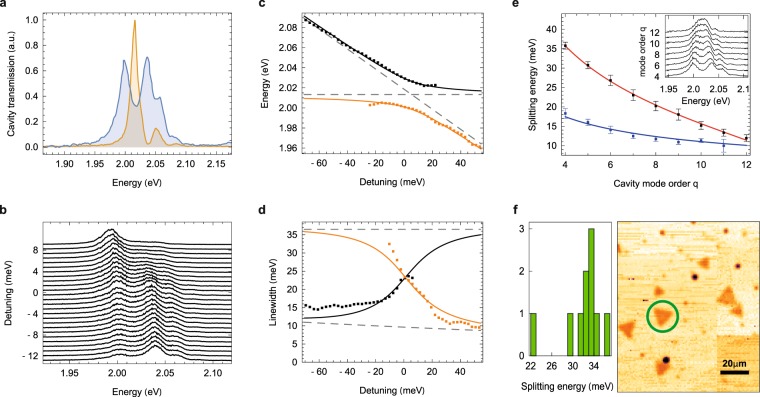
Polariton spectroscopy. (**a**) Bare cavity transmission spectrum (orange) and transmission spectrum under normal mode splitting at mode order *q* = 4 (blue). (**b**) Cavity transmission spectra of the *q* = 4 mode when tuned stepwise across the exciton resonance by varying the mirror separation. (**c**) Resonance energies of the upper (black) and lower (orange) polariton branches (data) derived from the spectra in **b**) and fits to the coupled oscillator model (solid lines; dashed lines show the empty cavity and bare exciton resonances). (**d**) Extracted linewidths of the upper (black) and lower (orange) polariton branches (data) and model fits (solid lines; the upper and lower dashed lines show the linewdiths of the empty cavity and the bare exciton resonances, respectively). (**e**) Polariton splitting $\hslash \Omega$ (upper data, fitted red line, see text) and coupling strength *g* (lower data and blue line) as a function of cavity mode order *q*. Error bars show the uncertainty of the fit used for evaluation. Example spectra for each mode order are shown in the inset, which are chosen at equal polariton peak height for better visibility. (**f**) Statistics of polariton splitting observed for 10 different flakes (left) and a map of cavity-enhanced sample extinction (right).

After these initial studies, we perform broadband transmission spectroscopy of the coupled cavity-TMD system at reduced intermirror distance to increase the cavity-flake coupling. For a mirror separation corresponding to the cavity mode order *q* = 4, Fig. 2a shows the cavity transmission spectra on (blue) and off (orange) the flake under white light illumination. The WS_2_ transmission spectrum exhibits a pronounced normal mode splitting with two well-resolved polariton resonances in response to the fundamental cavity mode, and weak higher-energy resonances due to transverse modes of the cavity. Tuning the cavity resonance across the emission spectrum by changing the mirror separation with a piezo actuator results in an avoided level crossing with spectra in Fig. 2b, which we compare to a coupled oscillator model in Fig. 2c,d.

The model accounts for the coupling with strength *g* between an optical cavity with the fundamental mode decay rate *κ*_0_ and an exciton transition with homogeneous linewidth *γ* to obtain the normal-mode or Rabi splitting Ω of the coupled oscillator system^28^:

1 $$\Omega (q)=2\sqrt{\sqrt{{g}^{4}+2{g}^{2}\gamma (\gamma +{\kappa }_{0}/q)}-{\gamma }^{2}}.$$

To model the detuning-dependent dispersion of the upper and lower polariton modes (see Supplementary Information) shown in Fig. 2c, we take *κ*_0_ = 51 meV and *γ* = 33 meV as determined from the initial characterization described above and use *g* as the only free fit parameter. Figure 2e shows the observed splitting and the deduced light-matter coupling strength *g* as a function of the longitudinal mode order *q*. For each *q* we observe and analyze an avoided level crossing as described above and shown in Fig. 2b–d to extract Ω on resonance where it is minimal for a given *q*. For better visibility, we show example spectra with equal polariton strength taken at slightly different detuning in the inset.

The Rabi splitting in Fig. 2e converges to Ω = 2*g* only for $g\gg (\gamma ,\kappa )$, which is approached for *q* = 4 where a maximal value of $\hslash \Omega$= 36 meV is observed, but drops to Ω ≈ *g* at large mirror separation where the two polariton branches merge. The splitting is smaller than reported in a similar experiment^11^ (with $\hslash \Omega$≈ 50 meV at *q* = 4), which can be traced back to a non-ideal PMMA layer thickness that reduces the local field at the sample in our experimental configuration. In principle, the PMMA layer could be removed to increase the coupling. We also note that we use a stable microcavity where excitons couple to a single, spectrally isolated cavity mode, while in ref.^11^ a planar Fabry-Perot displaying a mode continuum was used.

The model also accounts for the detuning dependence of the upper and lower polariton branch linewidths with Γ = (*κ* + *γ*)/2 on resonance (Fig. 2d). Since the cavity linewidth *κ* is significantly narrower than the exciton linewidth *γ*, coupling leads to a reduced polariton linewidth and thus to an increase of the polariton coherence lifetime compared to the ensemble exciton coherence by a factor of 1.7 on resonance, and up to a factor 4 at large detuning.

The observations of normal mode splitting and line narrowing as hallmarks of the strong-coupling regime are robust characteristics of the light-matter coupling for our sample. The right panel of Fig. 2f shows cavity-enhanced absorption measurements of other individual triangular WS_2_ monolayers. The left panel of Fig. 2f shows the statistics of resonant coupling experiments at *q* = 4 carried out on 14 of such flakes: while 10 flakes exhibited splitting energies in the range of $\hslash \Omega$ = 22–36 meV, four flakes did not exhibit signatures of strong coupling at all. For some of the latter flakes, lack of strong coupling can be explained by the presence of multiple WS_2_ layers, which we deduce from their higher absorption and lower fluorescence yield. Remarkably, most WS_2_ flakes in strong coupling featured comparable coupling strengths with a variation in *g* of only ±3 meV.

### Polariton hyperspectral imaging

**Figure 3:**
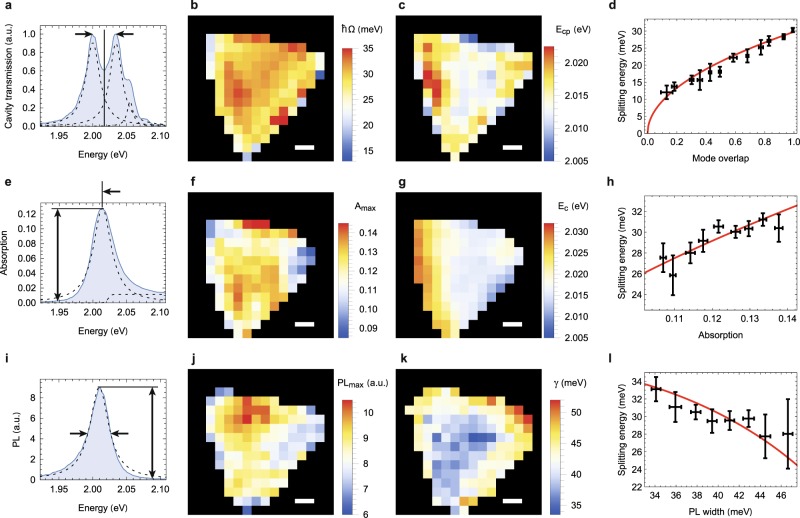
Polariton hyperspectral imaging. (**a**) Resonant cavity transmission showing a normal mode spectrum at *q* = 5. Spatial maps of (**b**) the Rabi splitting energy $\hslash \Omega$, and (**c**) the center energy of the normal mode spectrum *E*_cp_. (**d**) Dependence of $\hslash \Omega$ on the spatial mode overlap *η* with the flake. (**e**) Absorption spectrum at large *q* as inferred from cavity transmission spectroscopy. Maps of (**f**) the maximal resonant absorption *A*_max_, and (**g**) of the absorption center energy *E*_*c*_. (**h**) Correlation between $\hslash \Omega$ and the maximum resonant absorption, together with a fit (solid line, see text). (**i**) PL spectrum, (**j**) map of the peak PL intensity, and (**k**) PL linewdith. (**l**) Correlation between $\hslash \Omega$ and the PL linewidth together with a fit to Eq. 1 (solid line). The scale bar shows 2 *μ*m in all maps. All error bars correspond to standard deviations from datapoints that were binned.

Having established the signatures of strong coupling at individual spatial positions, we utilized the scanning capabilities of our cavity^20,21^ to study the two-dimensional nature of polariton landscapes in extended WS_2_ flakes. To this end, we stabilized the cavity length at *q* = 5 and ensure symmetric polariton populations as monitored in transmission spectroscopy. Under such conditions, the sample was displaced with respect to the cavity mode in 1 *μ*m steps corresponding to the spatial resolution determined by the cavity mode waist. At each raster-scan pixel we recorded white-light transmission spectra of the cavity as in Fig. 3a to obtain a spatial map of the polariton splitting, shown in Fig. 3b, and the center energy of the polariton doublet, shown in Fig. 3c.

To analyze the maps quantitatively, we first accounted for the spatial overlap between the cavity mode and the flake. Figure 3d shows how the observed Rabi splitting scales with the area overlap *η*. The dependence agrees very well with the expectation for collective coupling, where $g \sim \sqrt{\eta }$. To eliminate this overlap effect from further analysis, the map of Fig. 3b was corrected to show actual Rabi splitting values renormalized as $\Omega ={\Omega }_{raw}/\sqrt{\eta }$. Similar overlap corrections were performed for the PL and absorption maps in Fig. 3f,j, according to 1/*η*, and all data shown is limited to *η* > 0.35. The remaining deviations at edges are consistent with the notion of edges as line defects with light-matter characteristics distinct from the flake interior^29^.

It is instructive to confront the maps of polariton characteristics in the top panel of Fig. 3 with complementary parameters of light-matter coupling probed for the same flake with resonant absorption (central panel of Fig. 3) and PL (bottom panel of Fig. 3). Absorption spectra such as the one shown in Fig. 3e were recorded for a large mirror separation (~10 *μ*m) and used to evaluate the peak absorption (Fig. 3f) and its center energy (Fig. 3g) across the flake. For similar conditions, we also record the PL spectrum (Fig. 3i) and extracted the peak PL (Fig. 3j) and linewidth (Fig. 3k).

This comprehensive set of spatially-resolved measurements allows us to correlate external and internal properties of light-matter interaction in monolayer WS_2_. The polariton splitting in Fig. 3b shows a high degree of homogeneity with moderate variation across the flake, and a localized maximum along a line parallel to the left edge. This region is also apparent in a blue-shift of the polariton center energy *E*_*cp*_ (Fig. 3c) and also in the absorption center energy *E*_*c*_ (Fig. 3g).

The transition energy is sensitive to various internal and external parameters including strain^30–32^, doping^33^, or screening due to the dielectric environment^34–37^. Based on our data, strain is an unlikely explanation since it would correlate with the PL yield. However, we do not observe significant correlations between PL and *E*_*c*_. We also find no indication of a doping-induced energy shift, since no clear trion contribution was observed in the spectra^38^, even though part of the asymmetry of the fluorescence spectrum could originate from trions. However, trions do not contribute to absorption and thus do not affect polaritons^15^. The most probable scenario is that variations in the local dielectric environment are responsible for the inhomogeneities in light-matter coupling across the flake. The process used to transfer the flakes onto the mirror can lead to adsorbates such as water or KOH molecules located between the WS_2_ flake and the SiO_2_ surface, thereby introducing variations in the distance between the WS_2_ monolayer and the substrate. It is well known that the dielectric environment has an extraordinarily large impact on the excitonic properties of semiconductor monolayers, causing line shifts of up to hundreds of meV^34–37^. Our calculations show that variations of about 1 nm in the interlayer distance result in an energy shift of ~10 meV, consistent with our experimental observations (see Supplementary Information).

Next, we observe that the splitting $\hslash \Omega$ is clearly correlated with the resonant absorption map *A*_max_, see Fig. 3h. This can be well described by Eq. 1 when assuming an exciton oscillator strength that linearly depends on absorption, *f* ∝ *A*_max_, and a coupling strength that depends on *f* via $g=b\sqrt{f}$. We insert this expression into Eq. 1 and use *b* as a fit parameter to obtain the solid line shown in Fig. 3h. The agreement evidences that the observed variations in *A*_max_ are dominated by radiative excitonic transitions rather than quenching induced by defects. Notably, there is no apparent correlation between the integrated PL and the splitting. However, a clear correlation can be observed for the PL linewidth (and similarly for the absorption linewidth, not shown). As expected from Eq. 1, an anti-correlation between Ω and *γ* is expected. Originating either in excessive pure dephasing or inhomogeneous broadening, the increased linewidth towards the edges of the flake indicate an increased inhomogeneity and dissipation at these locations. Figure 3l shows good agreement between the observed linewidth dependence of the Rabi splitting and Eq. 1. Since the PL linewidth and the absorption center energy remain rather constant in the area where the absorption displays its largest variation, the different dependencies can be disentangled to a high degree, and the correlations shown in Fig. 3h,l) are dominated by a single quantity. Overall, we find that the variation in polariton splitting is governed by spatial variation of resonant absorption, inhomogeneous broadening and excessive pure dephasing or dissipation. With this we can link the polariton splitting to intrinsic exciton properties in a quantitative way.

### Spectroscopy of polariton-phonon coupling

**Figure 4:**
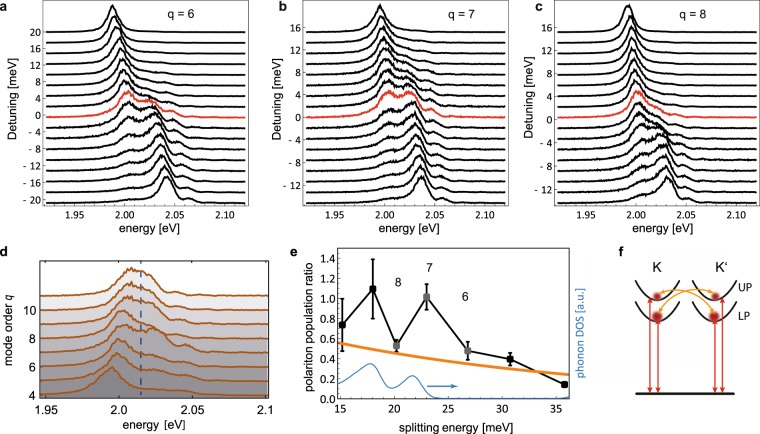
Polariton asymmetry due to phonon transitions. (**a**–**c**) Cavity transmission spectra for mode orders *q* = 6–8 for varying detuning from the exciton transition. Spectra with minimal normal mode splitting are indicated in red. The upper polariton at *q* = 7 yields larger population over the near-resonant detuning range compared to *q* = 6 and 8. (**d**) Normal mode spectra for different mode orders *q* = 4–11 with the cavity tuned to exact resonance with the exciton as inferred from avoided level crossings as in) (**a**–**c**) and Fig. 2c. (**e**) Polariton population ratio as a function of polariton splitting energy. The orange solid line shows the Boltzmann distribution, the blue solid line shows the phonon density of states adopted from ref.^40^. Error bars are uncertainties of *p*_*u*_/*p*_*l*_ as inferred from several fits to the data with different constraints. (**f**) Schematic level scheme of the ground state and two polariton states (UP, LP) for the K and K′ valleys with optical (red) and phonon (orange) transitions.

Finally, we turn to the influence of phonons on the polariton spectrum. It is well known from other systems that polariton-phonon coupling significantly affects polaritons^22,24^, and it was suggested that such coupling can lead to a surprising departure from the semi-classical behavior of the system^22,25^. For WSe_2_ it has been shown that a large exciton-phonon coupling strength can be present when optical transitions are in resonance with a phonon mode^39^. For our system, a directly observable effect is that the phonon bath mediates transitions between the dressed states and thereby alters the polariton population. In the experiment, we inspect the polariton population for exact cavity resonance conditions, i.e. where the polariton splitting is minimal for a given mode order *q*. Therefore, we analyze full avoided level crossing measurements as shown in Fig. 2b–d. Figure 4a–c show exemplary cavity transmission spectra under white light excitation for *q* = 6–8 for varying cavity detuning, where spectra at minimal polariton splitting are indicated in red. Figure 4d shows transmission spectra for resonant coupling for different *q*. A large asymmetry is apparent, which increases non-monotonically for decreasing cavity mode order. We evaluate the population ratio *p*_*u*_/*p*_*l*_ of the upper (UP) and lower (LP) polariton state from fits to the spectra and display the result as a function of the polariton splitting energy, see Fig. 4e. In this way, we can perform spectroscopy of the inter-polariton scattering strength within a single device at a fixed temperature. This is advantageous since the sample properties and the phonon bath remain fixed in this way. We find that the overall behavior is consistent with a thermal occupation following a Boltzmann distribution^24^ ${p}_{u}/{p}_{l}=\exp -\hslash \Omega /{k}_{B}T$ for *T* = 295 K (solid line in Fig. 4e, calculated with no free parameters). It is interesting to note that this Boltzmann factor is in stark contrast to the one observed in organic J-aggregate polaritons, where a thermalization governed by an energy gap $\Delta E\approx \hslash \Omega \mathrm{/2}$ is observed^22,24^, that can be ascribed to phonon scattering between uncoupled excitons and polaritons. Here, the presence of the full energy gap $\Delta E\approx \hslash \Omega$ suggests that direct scattering between the UP and LP polariton branch is the dominant process. Furthermore, we observe a significant deviation from thermalization in particular for mode order *q* = 7 ($\hslash \Omega =23$ meV) and *q* = 9 ($\hslash \Omega =18$ meV), where the two polariton branches remain essentially equally populated. The set of spectra shown in Fig. 4a–c) show that the larger population of the UP e.g. for *q* = 7 is visible across the entire near-resonant detuning range.

In principle, such a deviation could originate from a phonon bottleneck, resulting from a low phonon density of states at the relevant energy, such that thermalization is suppressed^24^. However, the splitting energy of mode *q* = 7 (*q* = 9) coincides with the energy of longitudinal acoustic (LA) (transverse acoustic(TA)) phonons at the K point with a significant density of states^40^ (blue solid line in Fig. 4e), ruling out the above interpretation. We also do not expect other processes such as polariton-electron scattering, exciton reservoir - polariton, or polariton-polariton scattering to be relevant for our experimental conditions, since we observe no signs of trions, find thermalization governed by the full UP-LP gap, and excite with sufficiently low powers such that the average polariton population remains much below one excitation within a cavity lifetime. Furthermore, the alternative pathway of phonon scattering of population selectively from the LP branch to a dark exciton state is an unlikely origin, since the bare energy gap of 47 meV between bright and dark exciton in the K and K’ valley^41^ is not consistent with the observed resonance energies.

A consistent explanation of the observed behaviour is the presence of direct resonant coupling between polaritons and LA or TA phonons, which establishes a non-thermal distribution via non-Markovian dynamics^22–24^, i.e. phonon-driven oscillations between UP and LP branches, which in the temporal average appear as the observed balanced polariton population ratio. The large density of states of K-point phonons furthermore suggests that transitions between polaritons states are accompanied by scattering between K and K’ valleys^42^ as depicted by the orange arrows in Fig. 4f. Further theoretical and experimental studies are required to substantiate this picture.

## Conclusion

In summary, we have introduced a technique that enables the simultaneous hyperspectral imaging of exciton polaritons, excitonic fluorescence, and absorption. The simultaneous measurement capabilities including extinction, photoluminescence, and polariton spectroscopy at different coupling strengths allows us to obtain comprehensive spectroscopic data from one and the same sample. We observe variations in the polariton splitting on a few-micron scale within a flake, as well as for different flakes. Clear correlations were established, interconnecting local variations in the oscillator strength and the exciton linewidth with the Rabi splitting. While the observed variations are already encouragingly small, further improvement in homogeneity is expected for flakes embedded in van der Waals heterostructures e.g. with hexagonal boron nitride. This could open the way for advanced polaritonic devices such as tunable polariton lasers and polariton Bose-Einstein condensation, and enable the realization of topological polaritons^17^. Furthermore, the observed deviation of the polariton population from a thermal distribution indicates a strong resonant interaction of polaritons and phonons, pointing towards non-Markovian dynamics. Such a scenario is expected to lead to superposition states involving polariton, valley, and phonon degrees of freedom^22^ with emergent quantum correlations^25^. In this way, control over light-matter interactions could be used to influence exciton-phonon coupling by all-optical means.

## Methods

### Cavity characterization

The mirrors were prepared by evaporating 50 nm silver as reflective layer and a SiO_2_ capping layer on top (20 nm at the fiber, 100 nm at the planar mirror) which protects silver from oxidation and serves as a spacer layer to place the sample at a field antinode. The fiber, shaped by CO_2_ laser machining^43^, has a conical tip to achieve smallest mirror separations^44^. In its center, we fabricate a concave profile with a radius of curvature of 75 *μ*m and a depth of 200 nm. We measure the cavity finesse ${\mathcal F}$ with a laser at 532 nm and obtain a value ${\mathcal F} =30$, and ${\mathcal F} =40$ from white light transmission spectra at the exciton emission energy *E*_0_ = 2.01 eV, in good agreement with a simulation. The planar mirror is mounted on a 3-axis nanopositioning stage (attocube ECS3030) to raster-scan the sample, while the fiber is mounted on a piezo actuator for fine tuning of the cavity length. To calibrate the optical cavity length, we record broad-band cavity transmission spectra with a supercontinuum laser and evaluate the separation of subsequent cavity resonances, see Fig. 1b. We find that the smallest accessible effective cavity length is *d*_eff_ = 4*λ*/2, corresponding to the longitudinal mode order *q* = 4, limited by the profile depth (200 nm) and the presence of the PMMA layer which covers the sample. At this separation, we obtain a cavity quality factor of ${Q}_{c}=q {\mathcal F} =200$. From scanning-cavity microscopy measurements and calculations, we infer the mode waist to be *w*_0_ = 1.0 *μ*m, which approximately defines the spatial resolution of the scanning cavity microscope. The finesse reduces for large mirror separation, but remains >35 over the range of *q* values shown in the manuscript. This is accounted for in the analysis.

### Sample preparation

WS_2_ monolayer crystals were grown by sulfurization of tungsten dioxide (WO_2_) powder. A SiO_2_/Si substrate along with a WO_2_ powder boat were placed at the center of a chemical vapor deposition (CVD) furnace. The SiO_2_/Si substrate was facing down in close proximity to the WO_2_ powder (99.99%, Sigma Aldrich). The temperature was initially ramped up rapidly but slowed down to 3 °C/min as it approached to 850 °C. Sulfur powder (99.5%, Alfa Aesar) was placed at upstream end of the quartz tube in a separate boat near the heating zone to allow vaporization (~110 °C) during the growth. The growth temperature was maintained for 15 minutes before cooling it down to the room temperature. 200 SCCM of Argon was used as a carrier gas during the entire process. As-grown monolayer crystals were studied in spectroscopy or transferred onto a mirror using established polymer-supported wet method. To this end polymethyl methacrylate (PMMA) was spin-coated on the monolayer flakes and lifted off in 1 M potassium hydroxide (KOH) in water. The PMMA-supported film with WS_2_ crystal flakes was rinsed in water for three cycles at room temperature to remove possible KOH residue and finally transferred onto mirror substrates.

### Photluminescence microscopy and spectroscopy

Excitation was performed either with a cw laser at 532 nm for PL or with a pulsed supercontinuum (Fianium Whitelase 450 SC, 20 MHz, ~50 ps) filtered to a spectral band of 580 nm to 650 nm. Confocal measurements were performed in a homebuilt setup including a 0.9 NA air objective. Detection was performed either with a Si avalanche photodetector or with a grating spectrometer (Princeton Instruments, Acton 2500) equipped with a sensitive CCD camera (Andor ikon-M). To observe the polariton spectrum we perform broad-band transmission spectroscopy of the coupled cavity-emitter system. We reduce the laser power such that during the pulse, much less than one photon populates the cavity on average to avoid multi-photon processes. The transmitted light was spectrally filtered, fiber coupled and recorded with the grating spectrometer.

### Model for environmental exciton energy renormalization

To assess the impact of variations in the distance between the optically active WS_2_ flake and the SiO_2_ substrate, we employ a multiscale approach introduced in ref.^37^. In a first step, an electrostatic model is used to determine an effective non-local dielectric function to account for screening of Coulomb interaction between carriers in the TMD in a vertical heterostructure environment. In a second step, band-structure renormalizations and the screened Coulomb potential enter calculations of the optical properties to determine the spectral positions of the excitonic resonances.

## Supplementary information

Supplementary Information
