# Supplementary material for: Polariton hyperspectral imaging of two-dimensional semiconductor crystals
Source: Sci Rep. 2019 Sep 24;9:13756. doi: 10.1038/s41598-019-50316-8 (PMC6760164; doi:10.1038/s41598-019-50316-8)
Supplement: Supplementary file 1 — Supplementary Information [file 41598_2019_50316_MOESM1_ESM.pdf]

Supplementary Information: Polariton hyperspectral imaging of two-dimensional semiconductor crystals

Christian Gebhardt,[1,2] Michael Förg,[3] Hisato Yamaguchi,[4] Ismail Bilgin,[4] Aditya D. Mohite,[4] Christopher Gies,[5] Malte Hartmann,[5] Matthias Florian,[5] Theodor W. Hänsch,[1,2] Alexander Högele,[3] and David Hunger[6,*]

[1] *Fakultät für Physik, Ludwig-Maximilians-Universität, Schellingstraße 4, 80799 München, Germany*
[2] *Max-Planck-Institut für Quantenoptik, Hans-Kopfermann-Str. 1, 85748 Garching, Germany*
[3] *Fakultät für Physik, Munich Quantum Center, and Center for NanoScience (CeNS) Ludwig-Maximilians-Universität, Geschwister-Scholl-Platz 1, 80539 München, Germany*
[4] *3MPA-11 Materials Synthesis and Integrated Devices, Materials Physics and Applications Division, Los Alamos National Laboratory (LANL), Los Alamos, New Mexico 87545, USA*
[5] *Institut für Theoretische Physik, Universität Bremen, 28334 Bremen, Germany*
[6] *Physikalisches Institut, Karlsruher Institut für Technologie, Wolfgang-Gaede-Str.1, 76131 Karlsruhe, Germany*

(Dated: September 20, 2018)

SETUP

Two laser sources can be coupled into the cavity fiber: a pulsed super-continuum source (SC) emitting 50 ps pulses at a rate of 20 MHz and a 532nm cw laser, which can be selected via a motorized mirror (MM). The fiber can be moved in z-direction with sub-nm precision via a piezo actuator, while the sample is mounted on an attocube nanopositioner (ECS3030) with three translational degrees of freedom. The collected light is split into two paths with a dichroic mirror (DM). The short wavelength part is detected with a photodiode (PD), the long wavelengths pass a notch filter (NF) and a longpass filter (LP) to be detected with a spectrometer consisting of different gratings and a CCD camera.

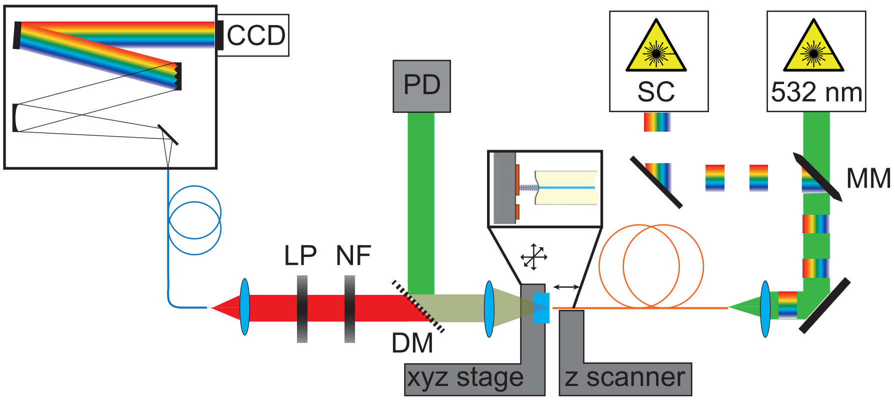

FIG. 1: Schematic drawing of the setup.

EVALUATION OF CAVITY TRANSMISSION SPECTRA

Cavity-enhanced absorption spectroscopy

We probe the transmission of broadband light from the SC source through the cavity with the grating spectrometer. We tune a set of cavity resonances stepwise across the spectrum and evaluate the resonances' peak transmission of multiple exposures to obtain a continuous spectrum. We perform measurements with a WS_2 flake located in the cavity mode (I_{flk}) and for an empty cavity as a reference (I_{ref}). We normalize the transmission spectrum to obtain the cavity-enhanced loss, $B_{\max} = 1 - I_{\text{flk}}/I_{\text{ref}}$, and calculate the peak absorption $A_{\max}$ from $B_{\max}$ and the mirror

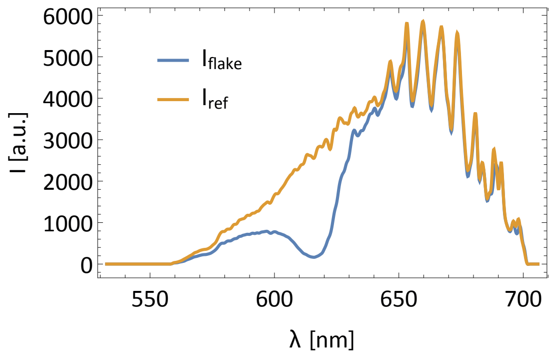

FIG. 2: Broadband cavity-enhanced transmission spectra taken for an empty cavity (orange) and with a WS_2 flake (blue).

reflectivity R as obtained from empty cavity measurements by solving the Fabry-Perot transmission function for $A_{\max}$,

$$A_{\max} = \frac{2B_{\max}(R(1-R) + (1-R^2) + \sqrt{(1-R^2)^2 - 4B_{\max}R(1-R)^2}}{2R^2(1-B_{\max})}. \tag{1}$$

This yields calibrated absorption spectra as shown in the manuscript.

Fitting of transmission spectra

We fit all spectra showing normal mode splitting with a triple Lorentzian, accounting for the two polariton resonances and one additional higher order transverse mode of the cavity associated with the upper polariton. The transversal mode of the lower polariton is neglected in the analysis, since its peak is not resolvable in the spectrum, as it is the case for other higher order modes. We expect an analogous anti-crossing behavior of the transversal mode as for the fundamental mode. From this we estimate that the transversal mode of the lower polariton contains less than 1% of the ground mode polariton intensities. We fit the following expression to the measured spectra:

$$S(\lambda) = a_{up}\frac{\Delta_{up}^2}{(\lambda-\lambda_{up})^2 - \Delta_{up}^2} + a_{lp}\frac{\Delta_{lp}^2}{(\lambda-\lambda_{lp})^2 - \Delta_{lp}^2} + a_{tm}\frac{\Delta_{tm}^2}{(\lambda-\lambda_{tm})^2 - \Delta_{tm}^2} \tag{2}$$

From the fits we extract the polariton wavelengths $\lambda_{up}, \lambda_{lp}$, the full-widths at half maximum of the polariton branches Δ_{up}, Δ_{lp}, and the amplitudes a_{up}, a_{lp}. Care is taken to obtain optimal fitting, and only reasonable fit values are used for later evaluations.

Polariton dispersion

The complex polariton eigenfrequencies are given by the following expression [1]:

$$\omega = \frac{\omega_{ex}+\omega_c}{2} - i\frac{\gamma+\kappa}{4} \pm \sqrt{g^2 + \frac{1}{4}\left(\omega_{ex} - \omega_c - i\frac{\gamma-\kappa}{2}\right)^2}.$$

The dispersion of the polaritons is given by the real part of the spectrum $Re(\omega)$. A simultaneous fit of the two branches to the dispersion data is performed to extract the coupling constant g and other parameters. We perform several fits and fix different parameters ((i) exciton and cavity linewidth, (ii) exciton energy and exciton and cavity linewidth, (iii) energy of zero detuning, exciton and cavity linewidth) in each case to obtain an estimate for the uncertainty of the fit.

Polariton width

The polariton linewidths are given by the imaginary part of the spectrum $-Im(\omega)$. A simultaneous fit of the linewidths to the two branches is performed to define the exciton linewidth γ. As for the dispersion relation, different

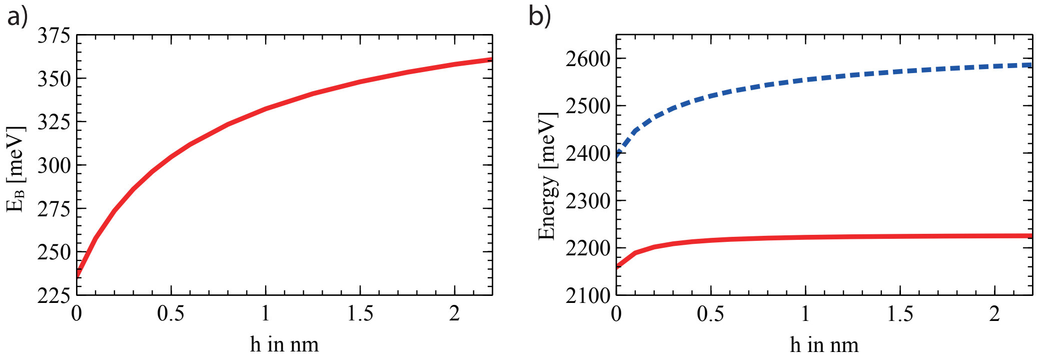

FIG. 3: a) Exciton binding energy as a function of the distance h between the WS_2 flake and the SiO_2 substrate. b) Resulting gap-dependent center energy of the excitonic absorption peak (red line) and electronic band gap (blue dotted line).

parameters are fixed ((i) cavity linewidth, (ii) exciton energy and cavity linewidth, (iii) energy of zero detuning and cavity linewidth) to check the robustness of the fits. From the models, we obtain an exciton linewidth of $\gamma \approx 37$ meV, in good agreement with confocal measurements and from cavity-enhanced fluorescence spectroscopy at large mirror separation.

Polariton intensity

We account for the wavelength dependence of the SC source and of the background absorption of the sample when evaluating the polariton intensities. Therefore, the transmission spectra are normalized by the SC spectrum as measured after the cavity without a sample to include wavelength dependent coupling efficiencies (see section above on cavity-enhanced absorption spectroscopy). In a second step, the effect of background absorption is corrected by normalizing with the wavelength dependent intracavity loss (not including the resonant absorption from the exciton). The normalized polariton intensities are then fitted with

$$p_{up,lp} = \Theta(\mp\delta)\cos\left[\frac{1}{2}\arctan\left(2\frac{g}{\delta}\right)\right]^2 + \Theta(\pm\delta)\sin\left[\frac{1}{2}\arctan\left(2\frac{g}{\delta}\right)\right]^2, \tag{3}$$

for the upper and lower polaritons.

THEORETICAL MODEL OF DIELECTRIC SCREENING

In Fig.3, we quantify the effect that local variations in the interlayer distance can have on the optical properties of a WS_2 monolayer between a PMMA layer and a silicon oxide substrate. Shown is the dependence on the distance between the TMD and the SiO_2 substrate, while the distance between PMMA and TMD is fixed at a realistic value of $5\AA$ [2]. The top panel shows the dependence of the band gap at the K point and the exciton binding energy. The resulting impact on the spectral position of the A-exciton transition is shown in the bottom panel. A variation of the gap to silicon oxide between 0.4 nm and 1.4 nm leads to a shift of the excitonic absorption peak of about 11 meV, which agrees with the variation observed in Fig. 3g in the main text.

* To whom correspondence should be addressed. E-mail: `david.hunger@kit.edu`

[1] Savona, V., Andreani, L. C., Schwendimann, P. & Quattropani, A. Quantum well excitons in semiconductor microcavities: Unified treatment of weak and strong coupling regimes. *Solid State Communications* **93**, 733–739 (1995).

[2] Rooney, A. P. *et al.* Observing imperfection in atomic interfaces for van der waals heterostructures. *Nano Lett.* **17**, 5222 (2017).
